# Spermatic Cord Knot: A Clinical Finding in Patients with Spermatic Cord Torsion

**DOI:** 10.1155/2011/310123

**Published:** 2011-11-29

**Authors:** Abdullatif Al-Terki, Talal Al-Qaoud

**Affiliations:** ^1^Urology Unit, Department of Surgery, Al-Amiri Hospital, Kuwait, Kuwait; ^2^Urology Residency Program, McGill University, Montreal, QC, Canada H3A 2T5

## Abstract

Pertinent history taking and careful examination often taper the differentials of the acute scrotum; congruently the ability to diagnose acute spermatic cord torsion (SCT) when radiological adjuncts are not available is highly imperative. This observational study serves to present a series of 46 cases of spermatic cord torsion whereby we hypothesize the identification of a clinical knot on scrotal examination as an important clinical aid in making a decision to surgical exploration in patients with acute and subacute SCT, especially in centers where imaging resources are unavailable.

## 1. Introduction

Reaching the confluence between clinical findings and imaging adjuncts remains a difficult task in diagnosing spermatic cord torsion (SCT) [1]. Awaiting a radiological diagnosis of SCT in a young patient with a high index of suspicion may lead to unnecessary delay especially in patients presenting in the intermediate stage of torsion, hence rapid assessment is mandatory, and salvaging the affected testis is the ultimate goal within the time window available and the present facilities. Previous studies have demonstrated loss of the cremasteric reflex to be 99% sensitive in patients with suspected torsion [2, 3], however, in the young patient in extreme pain and discomfort, eliciting such sign can be cumbersome.

**Figure 1:**
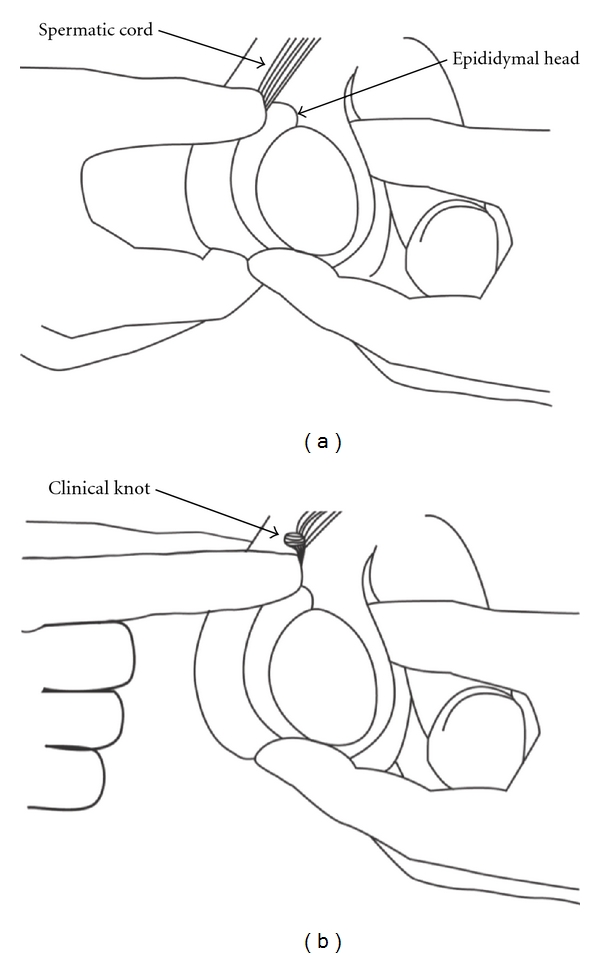
(a) Bimanual examination demonstrating normal findings. (b) Palpation at the junction of the spermatic cord and epididymal head whereby the clinical knot can be felt.

We present a series of cases, whereby upon clinical examination, SCT manifesting as a palpable cord knot distinct from the upper pole of the testes and epididymal head was observed, delineating the site of torsion of the cord: the spermatic cord knot. It is important to be able to demarcate the junction between the epididymal head and the cord where the knot will be felt. Following palpation of the testicle for lie, size, consistency, and to elicit tenderness, using a bimanual approach, the clinical knot is identified by starting at the epididymis and palpating its body up to the head, proceeding upward to palpate the spermatic cord for a semi-hard nodule, denoting the twisting of the cord (Figure 1).

## 2. Methods and Materials: Case Series

Available data from January 2009 to June 2011 on cases of acute scrotal pain presenting to our emergency department at Al-Amiri Hospital, Kuwait, was reviewed. Data on age (in years), duration of symptoms (in hours), site of pain, ultrasound use, presence of clinical knot on exam, and operative findings were extracted. The primary outcome was the presence of the spermatic cord knot on examination. Descriptive statistics of the series including frequency and percentages is presented stratified by diagnosis. Chi-squared test for trend tests was used to look for an association between age, site of torsion, the operative findings of degrees of rotation of the cord, and the primary outcome. Statistical analysis was conducted using STATA [4].

## 3. Results

In total, data was available on 114 patients (Table 1): 46 cases of suspected torsion (40%), 32 cases of epididymitits/orchitis (28%), 18 cases of varicocele (16%), 8 cases of inguinal hernia (7%), and 10 cases of undiagnosed pain (9%). The spermatic cord knot sign was seen amongst 40 (87% sensitivity) of the patients with SCT (Table 1), and amongst none of the other patients presenting with other diagnoses.

Amongst patients with SCT, the age range was 4–32 years (mean 18.3 years). The clinical knot sign was observed mostly in patients presenting in the early stage (1 to 7 hours) of SCT (1 to 7 hours: 74%, 7 to 24 hours: 22%, >24 hours: 4%). All patients with suspected SCT were taken for surgical exploration, 44 out of 46 (96%) patients were operated based on the clinical suspicion and finding of the cord knot on examination without the need for supplementary Doppler ultrasound. Most patients were operated within 2 hours of presentation, and contralateral orchiopexy was performed simultaneously; 4 cases (8%) had an unsalvageable testis (Table 2). Ultrasound was performed for two patients whom had presented at a late stage (>24 hours). Most patients had at least a 360-degree rotation of the testicle around its axis (45 patients, 98%, Table 2). Chi-squared test for trend demonstrated a significant association between degree of rotation and presence of clinical knot sign on examination (*P* = 0.006), however, chi-squared tests did not show an association between age (<16 versus >16 years) and site of torsion (right versus left) with presence of the clinical knot sign on examination (*P* = 0.81 and *P* = 0.55).

## 4. Discussion

Our modest series points to the potential aid the clinical knot sign adds to the emergency, pediatric, surgical, and urological staff attending to the case of acute scrotum presenting in the acute and subacute stage, when imaging is unavailable or delays action. Diagnosing SCT can be difficult, and distinction of the scrotal contents is necessary while paying particular attention to identifying the epididymis and delineating the cord from the epididymal head. A common clinical diagnostic dilemma in patients with acute scrotal pain is the inability to differentiate SCT from epididymitis and/or orchitis [5]. We demonstrated that one could help differentiate that by identifying clinical knot sign that was not present in other cases of acute scrotal pain, without delaying surgical exploration.

Earlier, MR imaging has shown specific signs that help differentiate SCT from epididymitis: the whirpool/twisting pattern and the torsion knot, which appear as swirls centered over a low-signal-intensity focus [6]. Later, a report on two cases was published demonstrating similar findings on sonography [7], whereby a central echogenic focus was seen correlating to the low-signal-intensity focus seen on MR equivalent to the torsion knot. Although previous reports have demonstrated the identification of the whirlpool pattern and torsion knot [6–10], previous literature has not approached this sign on clinical examination. Despite Doppler ultrasound having a high sensitivity and specificity [11] in detecting testicular torsion with blood flow patterns to help delineate torsion from inflammation (epididymitis/orchitis), and alternative techniques such as scintigraphy and MR imaging achieving even higher diagnostic accuracy [12], the use of imaging as an adjunct may only be justified in patients with a low suspicion of acute SCT. Ultrasound was used in our series as an adjunct only for 2 patients with SCT, whom presented in the late stage whereby further delay awaiting imaging would cause no further harm than already present.

Since our aim from this observational study, based on a case series, was to emphasize on a clinical finding, the spermatic cord knot, as a potential adjunct to ultrasound and imaging in centers where these facilities are unavailable, inherently our description lacks comprehensive statistical analysis. In an attempt, our results demonstrate that amongst those without a positive sign on exam, the clinical knot was still evident on surgical exploration, pointing to the difficulty that can be faced in eliciting such sign, and yet a considerably high sensitivity of 86%, and a very low specificity. However, this must be weighed against the small series presented and the fact that all patients taken for surgical exploration had underlying torsion, that is, no true negatives to serve as a numerator for a predictive value of a negative examination. As one would expect, our analysis shows a significant association between degrees of rotation of the testicle around its cord and the presence of knot on examination, however, no association was found between age and site of torsion with presence of the knot on examination.

## 5. Conclusion

We claim the identification of the clinical knot sign on examination helps to reassure the examining doctor of his/her suspicion of SCT in the acute and subacute stage, most importantly avoiding delay in awaiting imaging findings and decision to surgical exploration. The description of this clinical sign is particularly important to rural centers of limited resources, and in centers where Doppler and MRI studies are not readily available to aid diagnosis. However, as a result of the small number of cases, an inherent limitation of this descriptive series is our inability to reach a firm inference yet, and despite advocating the identification of this sign as a strong suspicion to proceed to scrotal exploration, a larger prospective study would enrich statistical power and serve to calculate more robust estimates of incidence, sensitivity, and specificity, and further facilitating exploration of factors associated with the spermatic cord knot while simultaneously accounting for possible confounders.

## Figures and Tables

**Table 1:** Descriptive statistics of patients stratified by diagnosis.

	Diagnosis				
	Torsion, *N* (%) 46 (40)	Epididymorchitis, *N* (%) 32 (28)	Varicocele, *N* (%) 18 (16)	Hernia, *N* (%) 8 (7)	Unexplained pain, *N* (%) 10 (9)
Age (yrs)					
4–16	18 (39)	4 (13)	6 (33)	1 (13)	3 (30)
>16	28 (61)	28 (87)	12 (67)	7 (87)	7 (70)
Duration of symptoms (hours)					
1 to 7	34 (74)	3 (9)	0 (0)	0 (0)	0 (0)
>7 to 24	10 (22)	5 (15)	2 (11)	1 (13)	0 (0)
>24	2 (4)	24 (75)	16 (81)	7 (87)	10 (0)
Site					
Right	21 (46)	16 (50)	2 (11)	5 (63)	1 (10)
Left	25 (54)	12 (38)	10 (56)	3 (37)	6 (60)
Bilateral	0	4 (12)	6 (33)	0 (0)	3 (30)
Ultrasound used	2 (4)	32 (100)	18 (100)	∗	10 (100)
Positive clinical knot sign	40 (87)	0 (0)	0 (0)	0 (0)	0 (0)

*N*: number of patients, ∗ referred to general surgery.

**Table 2:** Clinical and operative findings of patients with spermatic cord torsion.

	Right sided torsion *N* = 21	Left sided torsion *N* = 25	*N* (%)
Pre-op clinical knot sign positive	18	22	40 (87)
Positive intraoperative sign	21	25	46 (100)
Degree of rotation			
180	0	1	1 (2)
360	4	8	12 (26)
>360	17	16	33 (72)
Salvage of testicle	20	22	42 (91)
Orchiectomy	1	3	4 (9)
